# Association of haemoglobin glycation index with outcomes in patients with acute coronary syndrome: results from an observational cohort study in China

**DOI:** 10.1186/s13098-022-00926-6

**Published:** 2022-10-31

**Authors:** Jiayu Li, Yanguo Xin, Jingye Li, Li Zhou, Hui Qiu, Aidong Shen, Hui Chen, Hongwei Li

**Affiliations:** 1 Department of Cardiology, Beijing Friendship Hospital, Capital Medical University. No, 95 Yong’an Road, Xicheng District, Beijing, 100050 China; 2 Department of Internal Medical, Medical Health Center, Beijing Friendship Hospital, Capital Medical University, Beijing, China; 3 Beijing Key Laboratory of Metabolic Disorder Related Cardiovascular Disease, Beijing, China

**Keywords:** Hemoglobin glycation index, Acute coronary syndrome, Major adverse cardiac and cerebrovascular events, China

## Abstract

**Background:**

The hemoglobin glycation index (HGI) is the difference between measured and estimated glycation of hemoglobin. However, there is limited evidence to investigate the HGI and the clinical outcomes of acute coronary syndrome patients. This study aimed to evaluate the association between HGI and the clinical outcomes of acute coronary syndrome (ACS) in a China cohort.

**Method:**

This single-center retrospective study was carried out in the Cardiovascular Center of Beijing Friendship Hospital, a total of 11004 consecutive patients with ACS from Dec 2012–Dec 2020 were enrolled in this study. Patients were divided into quintiles according to their HGI levels. The incidence of major adverse cardiac and cerebrovascular events (MACCEs) was recorded.

**Result:**

HGI were divided into five quintiles quintiles: −0.906 (−7.188, −0.663), −0.491 (−0.663, −0.343), −0.196 (−0.342, −0.039), 0.170 (−0.039, 0.485), and 1.156 (0.485, 7.875), respectively.

Competing risk regression revealed that HGI was positively related to all-cause death, CV death, and composite MACCEs. Multivariate Cox proportional hazards regression analysis indicated that hypertension (HR:1.109, *P* = 0.013), previous stroke (HR:1.208, *P* < 0.001), past PCI (HR: 1.268, *P* < 0.001), age (HR: 1.011, *P* < 0.001), BMI (HR: 0.987, *P* = 0.012), heart rate (HR: 1.004, *P* = 0.001), NSTEMI (HR: 1.205, *P* < 0.001), WBC (HR: 1.020, *P* = 0.008), eGFR (HR: 0.993, *P* < 0.001), HDL-C (HR: 0.809, *P* = 0.002), LVEF (HR:0.240, *P* < 0.001), LM/three-vessel or proximal LAD involved (HR: 1.208 *P* < 0.001; HR:0.914, *P* = 0.019, respectively), and antiplatelet agents during hospitalization (HR:0.806, *P* = 0.020) independently predicted the incidence of MACCEs in ACS patients. Restricted cubic spline indicated a U-shaped association between the HGI and risk of MACCEs.

**Conclusion:**

Both low HGI and high HGI was associated with an increased risk of adverse outcomes in patients with acute coronary syndrome, compared with moderate HGI.

**Supplementary Information:**

The online version contains supplementary material available at 10.1186/s13098-022-00926-6.

## Introduction

Acute coronary syndrome (ACS) is the leading cause of death worldwide [1]. Diabetes mellitus (DM) is considered to confer equal risk to that of coronary artery disease for cardiovascular mortality [2, 3]. According to recent evidence, approximately 20–25% of patients with ACS reportedly also have DM [4]. A large body of evidence indicates that proper glucose control contributes to long-term cardiovascular benefits. Patients with ACS and DM suffer higher mortality than those with only ACS [5, 6].

Haemoglobin A1c (HbA1c) is an indicator reflecting the glucose level over the past 3 months and has been the most commonly used marker of glucose control [7]. The latest guideline for diabetes recommended HbA1c > 6.5% as a diagnostic criterion for diabetes [8]. However, as HbA1c is an important parameter of average glucose levels, and there is a significant linear correlation between blood glucose and HbA1c, there are interindividual variations in the rate of haemoglobin glycation caused by factors other than mean blood glucose levels among patients with or without diabetes [9, 10]. In addition, some studies found that only 60–80% of patients demonstrated consistent HbA1c and average blood glucose levels [11]. various factors such as glucose metabolism, genetic factors and passive hemoglobin glycation rates affected HbA1c largely. Recently, a new indicator, haemoglobin glycation index (HGI), was introduced to quantify this variation [12].

HGI is defined as the disparity between the observed and predicted HbA1c according to a linear regression between HbA1c and fasting plasma glucose (FPG) [13]. Some evidence has investigated the clinical applications of HGI in diabetes. Daiji and colleagues [14] reported a positive correlation between HGI and systemic arterial stiffening independent of hyperglycaemia. Another study from Yuesong presented a U-shaped relationship between HGI and the risk of diabetic patients with stroke, indicating that both low and high HGI is associated with a higher risk of poor prognosis [15]. However, there are rare clinical studies evaluating the applications of HGI among ACS patients.

This study aimed to assess the association between HGI and the prognosis in ACS subjects with or without diabetes using single-centre cohort data.

## Methods

### Study population

**Fig. 1:**
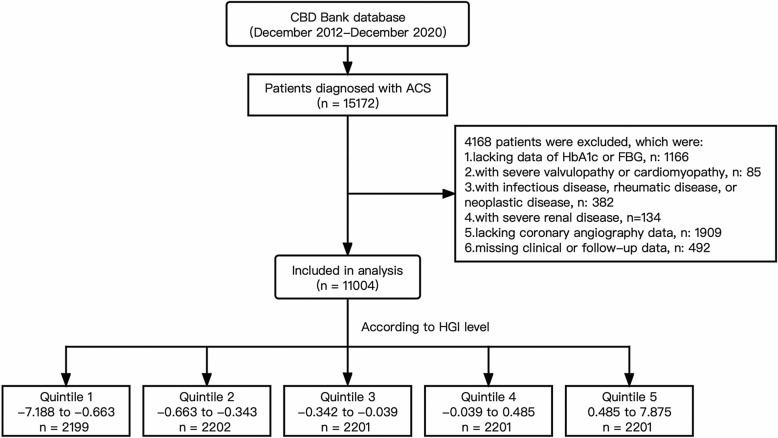
Flow chart of study subject enrollment. *CBD* Cardiovascular Center of Beijing Friendship Hospital Database, *ACS* acute coronary syndrome, *CAG* coronary angiography; *HbA1c* haemoglobin A1c, *FPG* fast plasma glucose, *HGI* haemoglobin glycation index

All participants were included from the Cardiovascular Center of Beijing Friendship Hospital. A total of 15172 consecutive patients diagnosed with ACS from Dec 2012–Dec 2020 were enrolled. According to the flow chart (Fig. 1), 4168 patients were excluded according to the exclusion criteria: (1) 1166 patients lacked HbA1c or FBG data, (2) 85 patients were diagnosed with severe valvular diseases or cardiomyopathy, (3) 382 patients were suffering from infectious disease, rheumatic disease, or neoplastic disease, (4) 134 patients were diagnosed with severe renal disease, (5) 1909 patients lacked coronary angiography data, and (6) 492 patients had missing clinical or follow-up data. The final follow-up ends up at Dec 31, 2021, with a median follow-up of 36.5 months. All enrolled patients were followed-up by phone interview, clinical visiting, or hospital records.

### Data collections and definitions

This study was approved by the ethics committee of Beijing Friendship Hospital and conducted in accordance with the Declaration of Helsinki.

**Fig. 2:**
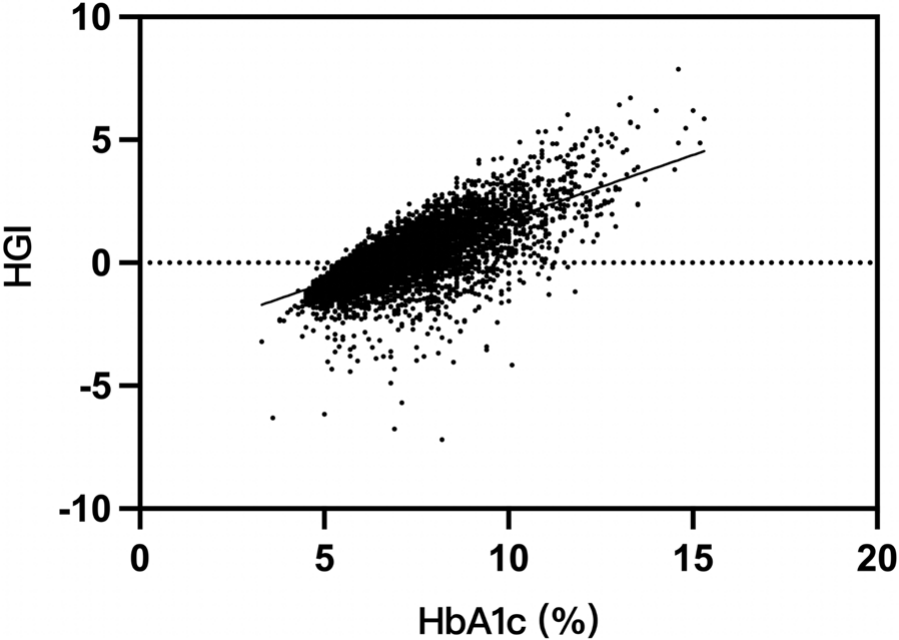
The correlation of HbA1c and the calculated HGI by subtracting the predicted HbA1c from the observed HbA1c. *HbA1c* haemoglobin A1c, *HGI* haemoglobin glycation index

All the basic information was recorded by two independent persons. The concentrations of fasting plasma glucose (FPG) and HbA1c was tested after hospitalization at the clinical laboratory of our hospital. Predicted HbA1c was calculated by inserting the corresponding FPG value into the linear regression equation (HbA1c [%] = 4.036 + 0.399 FPG [mmol/L], *P* < 0.001, adjusted r = 0.691). HGI was the difference between the predicted HbA1c and the observed HbA1c, the correlation is shown (Fig. 2). The population was then divided into five quintiles according to their HGI levels. Major adverse cardiac and cerebral events were recorded during follow-up periods.

Criteria for diabetes mellitus included (1) currently receiving anti-diabetic medication, (2) typical diabetic symptoms plus an FPG ≥ 7.0 mmol/L, (3) typical diabetic symptoms with random blood glucose (RBG) ≥ 11.1 mmol/L, and (3) a positive oral glucose tolerance test (OGTT) (2-h plasma glucose level ≥ 11.1 mmol/L). Hypertension was defined as currently receiving antihypertensive agents or blood pressure equal to or greater than 140/90 mmHg three times on different days. The criteria for dyslipidaemia were identified as one of the following conditions: (1) fasting total cholesterol (TC) > 5.18 mmol/L, (2) low-density lipoprotein cholesterol (LDL-C) > 3.36 mmol/L, (3) high-density lipoprotein cholesterol (HDL-C) < 1.03 mmol/L, (4) triglyceride (TG) > 1.69 mmol/L, and (5) previous use of lipid-lowering agents. Acute coronary syndrome included ST-elevation myocardial infarction (STEMI), non-ST-segment elevation myocardial infarction (NSTEMI), and unstable angina (UA).

MACCEs include all-cause death (including CV death and non-CV death), CV death, nonfatal MI, cardiac rehospitalization (rehospitalization due to heart failure or ACS), revascularization, and nonfatal stroke (ischaemic and haemorrhagic stroke). Nonfatal stroke was identified as neural dysfunction due to vascular sudden rupture or obstruction and diagnosed according to the signs of brain dysfunction or imaging evidence.

### Statistical analyses

Mean ± standard deviation (SD) or median (IQR) were applied to present continuous variables. Student’s test or Mann–Whitney U-test were used to analyze the comparisons among these groups. Categorical variables were shown as numbers and percentages, which were compared via the Pearson chi-square test or Fisher’s exact test. First, Kaplan–Meier plots were generated to estimate the cumulative incidence of the outcomes in different HGI groups. To figure out the factors related to MACCEs, then, baseline variables were enrolled to correlate with MACCEs by univariate analysis, and the related factors entered the multivariate model with the use of Cox hazards regression models. Considering the competitive risk between all-cause death and other endpoints, Competing risk model was employed to analyze the incidence of MACCEs in different HGI levels and evaluate the predictive effect of the HGI on clinical outcomes. To further examine the continuous association between HGI levels and mortality, Restricted cubic spline (RCS) was used to analyze the relationship between HGI value and types of MACCEs. Subgroup analyses were also performed according to the hazard ratios of MACCEs after adjusting for confounding factors. Statistical tests were performed with SPSS statistics 26, Stata MP 15.1, and the R Programming Language. A two-tailed *P* value < 0.05 was considered as statistically significant.

## Results

### Baseline characteristics of patients

The baseline characteristics of the enrolled patients in different HGI groups are illustrated (Table 1). The median HGI of the 11004 patients was −0.196 (−7.188, 7.875). The five quintiles are −0.906 (−7.188, −0.663), −0.491 (−0.663, −0.343), −0.196 (−0.342, −0.039), 0.170 (−0.039, 0.485), and 1.156 (0.485, 7.875), respectively. In the Q2-Q3 groups, the prevalence of diabetes, dyslipidaemia, and stroke were significantly lower than those in the Q1, Q4 and Q5 groups. The systolic blood pressure and medication usage on admission (antiplatelet agents, ACEIs/ARBs, beta-blockers, and statins) increased with the HGI levels. In addition, BMI, HbA1c, and FPG are positively associated with HGI. Male patients are more likely to have a lower HGI.

**Table 1:** Baseline characteristics of the study population

Variable	Total population	Quintile 1	Quintile 2	Quintile 3	Quintile 4	Quintile 5	*p* value
	n = 11,004	n = 2199	n = 2202	n = 2201	n = 2201	n = 2201	
Median HGI (range)	−0.196 (−7.188, 7.875)	−0.906 (−7.188, −0.663)	−0.491 (−0.663, −0.343)	−0.196 (−0.342, −0.039)	0.170 (−0.039, 0.485)	1.156 (0.485, 7.875)	< 0.001
Age, years	64.88 ± 10.92	62.59 ± 11.45	64.10 ± 10.97	65.71 ± 10.58	66.61 ± 10.446	65.40 ± 10.71	< 0.001
Male gender	7227 (65.7%)	1640 (74.6%)	1549 (70.3%)	1381 (62.7%)	1342 (61.0%)	1315 (59.7%)	< 0.001
Medical history							
Current/ex-Smoker	6220 (56.5%)	1372 (62.4%)	1292 (58.7%)	1181 (53.7%)	1174 (53.3%)	1201 (54.6%)	< 0.001
Hypertension	7775 (70.7%)	1442 (65.6%)	1503 (68.3%)	1527 (69.4%)	1647 (74.8%)	1656 (75.2%)	< 0.001
Diabetes	4157 (37.8%)	527 (24.0%)	390 (17.7%)	515 (23.4%)	961 (43.7%)	1764 (80.1%)	< 0.001
Dyslipidemia	5216 (47.4%)	999 (45.4%)	985 (44.7%)	1045 (47.5%)	1084 (49.3%)	1103 (50.1%)	0.001
Previous Stroke	1899 (17.3%)	321 (14.6%)	358 (16.3%)	379 (17.2%)	392 (17.8%)	449 (20.4%)	< 0.001
Previous MI	1122 (10.2%)	179 (8.1%)	217 (9.9%)	196 (8.9%)	246 (11.2%)	284 (12.9%)	< 0.001
Past PCI	1589 (14.4%)	221 (10.1%)	281 (12.8%)	300 (13.6%)	359 (16.3%)	428 (19.4%)	< 0.001
Past CABG	252 (2.3%)	23 (1.0%)	44 (2.0%)	37 (1.7%)	65 (3.0%)	83 (3.8%)	< 0.001
Physical examination							
BMI, kg/m2	25.80 ± 3.59	25.60 ± 3.50	25.71 ± 3.62	25.69 ± 3.65	25.85 ± 3.60	26.14 ± 3.57	< 0.001
SBP, mmHg	131.43 ± 19.37	129.71 ± 20.77	130.38 ± 18.76	131.25 ± 18.24	132.70 ± 19.24	133.11 ± 19.55	< 0.001
DBP, mmHg	75.35 ± 11.89	75.72 ± 12.86	75.37 ± 11.77	75.08 ± 11.40	75.51 ± 11.72	75.08 ± 11.65	0.517
Heart rate, bpm	72.19 ± 13.14	73.83 ± 14.48	71.76 ± 12.90	70.46 ± 12.00	71.36 ± 12.81	73.54 ± 13.10	< 0.001
Clinical presentation							
STEMI	1950 (17.7%)	715 (36.7%)	394 (20.2%)	305 (15.6%)	266 (13.6%)	270 (13.8%)	< 0.001
NSTEMI	1890 (17.2%)	420 (22.2%)	364 (19.3%)	338 (17.9%)	358 (18.9%)	410 (21.7%)	< 0.001
UAP	7164 (65.1%)	1064 (14.9%)	1444 (20.2%)	1558 (21.7%)	1577 (22.0%)	1521 (21.2%)	< 0.001
Medication on admission							
Antiplatelet agent	4002 (36.4%)	668 (30.4%)	791 (35.9%)	832 (37.8%)	855 (38.8%)	856 (38.9%)	< 0.001
ACEI/ARB	3777 (34.3%)	624 (28.4%)	673 (30.6%)	772 (35.1%)	851 (38.7%)	857 (38.9%)	< 0.001
Beta-blocker	2443 (22.2%)	385 (17.5%)	474 (21.5%)	495 (22.5%)	562 (25.5%)	527 (23.9%)	< 0.001
Statins	3434 (31.2%)	513 (23.3%)	640 (29.1%)	725 (32.9%)	766 (34.8%)	790 (35.9%)	< 0.001
Laboratory data							
WBC, 10^9^/L	7.20 ± 2.46	7.74 ± 2.98	7.08 ± 2.52	6.91 ± 2.24	6.97 ± 2.08	7.29 ± 2.28	< 0.001
Hemoglobin, g/L	135.00 ± 11.00	138.00 ± 24.00	137.00 ± 21.00	134.00 ± 21.00	133.00 ± 23.00	133.00 ± 24.00	< 0.001
Hs-CRP, mg/L	2.39 ± 4.25	2.99 ± 9.55	2.15 ± 5.77	2.10 ± 5.82	2.28 ± 5.72	2.63 ± 5.81	< 0.001
RBG at admission, mmol/L	7.63 ± 2.07	7.26 ± 2.95	6.83 ± 2.91	6.91 ± 3.14	7.85 ± 3.44	10.63 ± 6.14	< 0.001
FPG, mmol/L	5.54 ± 1.35	5.77 ± 2.19	5.21 ± 1.06	5.16 ± 1.24	5.50 ± 1.85	6.94 ± 3.58	< 0.001
HbA1c,%	6.10 ± 0.90	5.40 ± 0.70	5.60 ± 0.50	5.90 ± 0.50	6.40 ± 0.80	8.10 ± 2.00	< 0.001
Albumin, g/L	38.75 ± 3.97	38.66 ± 4.09	38.84 ± 3.80	38.92 ± 3.88	38.88 ± 3.97	38.46 ± 4.10	0.217
Creatinine, umol/L	87.36 ± 70.73	90.97 ± 76.07	85.52 ± 61.95	83.41 ± 53.69	90.45 ± 86.61	86.44 ± 70.51	< 0.001
eGFR, ml/min/1.73m^2^	82.91 ± 22.32	83.80 ± 23.25	84.09 ± 20.86	82.89 ± 20.75	81.01 ± 22.69	82.75 ± 23.76	0.001
TC, mmol/L	4.28 ± 1.06	4.33 ± 1.01	4.28 ± 1.04	4.33 ± 1.07	4.22 ± 1.05	4.24 ± 1.11	< 0.001
TGs, mmol/L	1.37 ± 0.36	1.39 ± 0.97	1.32 ± 0.90	1.34 ± 0.93	1.36 ± 0.91	1.45 ± 1.07	< 0.001
LDL-C, mmol/L	2.43 ± 0.76	2.47 ± 0.74	2.43 ± 0.76	2.44 ± 0.77	2.38 ± 0.75	2.41 ± 0.78	0.001
HDL-C, mmol/L	1.08 ± 0.26	1.08 ± 0.27	1.09 ± 0.27	1.11 ± 0.27	1.08 ± 0.26	1.04 ± 0.25	< 0.001
Echocardiography							
LVEF	0.65 ± 0.05	0.64 ± 0.11	0.66 ± 0.09	0.66 ± 0.07	0.66 ± 0.08	0.65 ± 0.09	< 0.001
Angiography findings							
LM/three-vessel	5995 (54.5%)	1161 (52.8%)	1133 (51.5%)	1091 (49.6%)	1209 (54.9%)	1401 (63.7%)	< 0.001
Proximal LAD	4688 (42.6%)	944 (42.9%)	935 (42.5%)	896 (40.7%)	926 (42.1%)	987 (44.8%)	0.087
Medication during hospitalization							
PCI/CABG	6229 (56.6%)	1352 (61.5%)	1205 (54.7%)	1172 (53.2%)	1182 (53.7%)	1318 (59.9%)	< 0.001
Antiplatelet agent	10,317 (93.8%)	2060 (93.7%)	2064 (93.7%)	2074 (94.2%)	2071 (94.1%)	2048 (93.0%)	0.528
ACEI/ARB	6075 (55.2%)	1234 (56.1%)	1171 (53.2%)	1158 (52.6%)	1243 (56.5%)	1269 (57.7%)	0.002
Beta-blocker	7509 (68.2%)	1519 (69.1%)	1482 (67.3%)	1426 (64.8%)	1495 (67.9%)	1587 (72.1%)	< 0.001
Statins	9907 (90.0%)	1932 (87.9%)	2002 (90.9%)	1994 (90.6%)	1992 (90.5%)	1987 (90.3%)	0.005
Hypoglycemic agents							
Alpha-glucosidase inhibitor	2529 (23.0%)	316 (14.4%)	224 (10.2%)	290 (13.2%)	592 (26.9%)	1107 (50.3%)	< 0.001
Metformin	1514 (13.8%)	136 (6.2%)	129 (5.9%)	171 (7.8%)	360 (16.4%)	718 (32.6%)	< 0.001
Sulfonylurea	857 (7.8%)	95 (4.3%)	54 (2.5%)	100 (4.5%)	202 (9.2%)	406 (18.4%)	< 0.001
DPP-4i	31 (0.3%)	3 (0.1%)	2 (0.1%)	2 (0.1%)	5 (0.2%)	19 (0.9%)	< 0.001
Insulin	924 (8.4%)	114 (5.2%)	54 (2.5%)	62 (2.8%)	129 (5.9%)	565 (25.7%)	< 0.001
Insulin sensitizer	267 (2.4%)	32 (1.5%)	22 (1.0%)	30 (1.4%)	60 (2.7%)	123 (5.6%)	< 0.001

*HGI* hemoglobin glycation index, *MI* myocardial infarction, *PCI* percutaneous coronary intervention, *CABG* Coronary Artery Bypass Grafting, *BMI* body mass index, *SBP* systolic blood pressure, DBP diastolic blood pressure, *STEMI*ST-elevated myocardial infarction, *NSTEMI* non-ST elevated myocardial infarction, *UAP* unstable angina pectoris, *ACEI* angiotensin-converting enzyme inhibitors, ARB angiotensin receptor blockers, *WBC* white blood cells, *Hs-CRP*Hypersensitive c-reactive protein, *RBG* random blood glucose, *FPG* fast plasma glucose, *eGFR* estimated glomerular filtration rate, *TC* total cholesterol, *TGs* triacylglycerol, *LDL-C* low density lipoprotein cholesterol, *HDL-C* high density lipoprotein cholesterol, LVEF left ventricular ejection fraction, *LM* left main vessel, *LAD* left anterior descending artery, *DPP-4i* dipeptidyl peptidase-4 inhibitors

### HGI predicted the occurrence of MACCEs

**Fig. 3:**
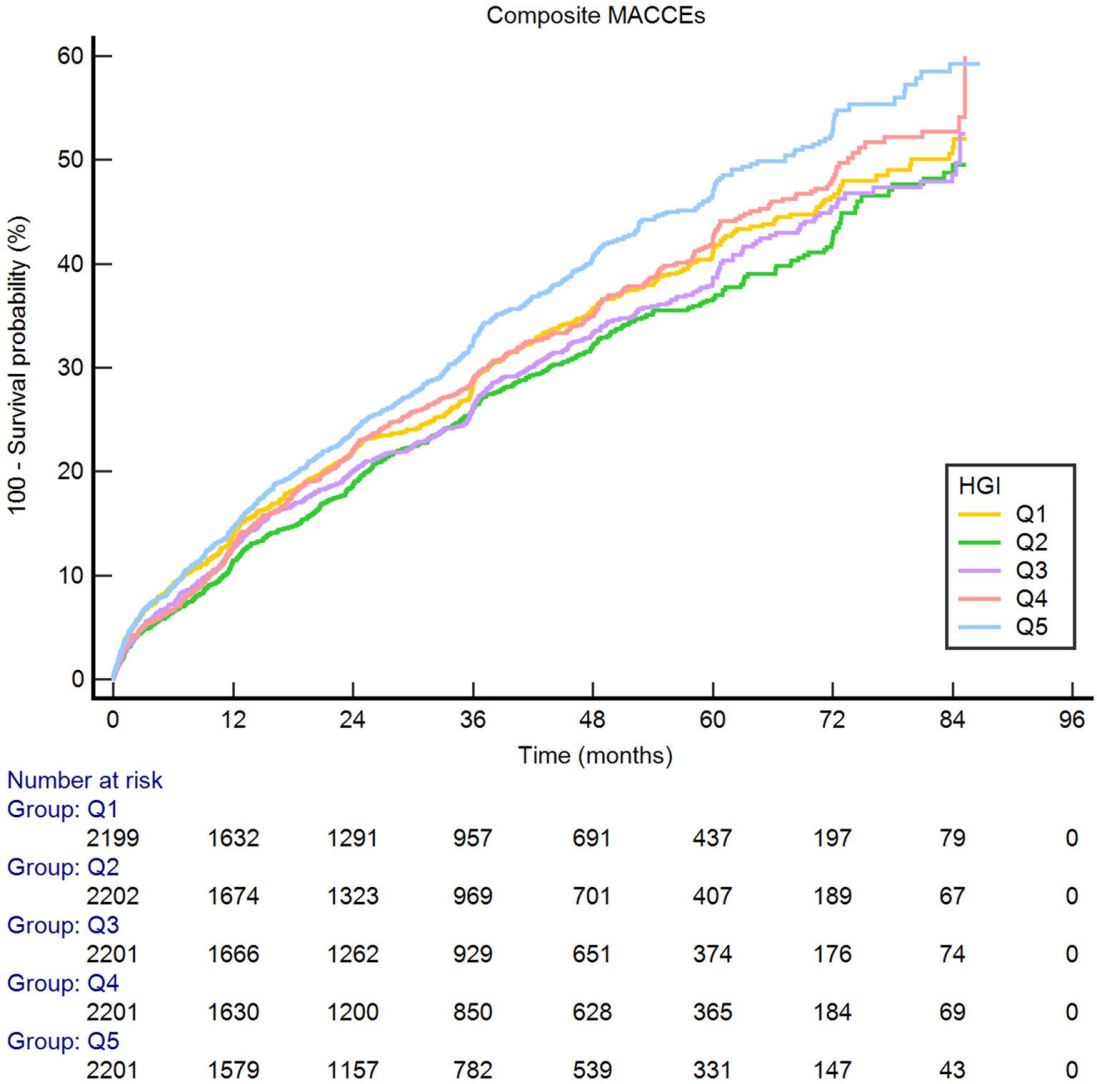
Kaplan–Meier curves for composite MACCEs of the the five quintiles. *MACCEs* major adverse cardiac and cerebral events, *HGI* haemoglobin glycation index

**Fig. 4:**
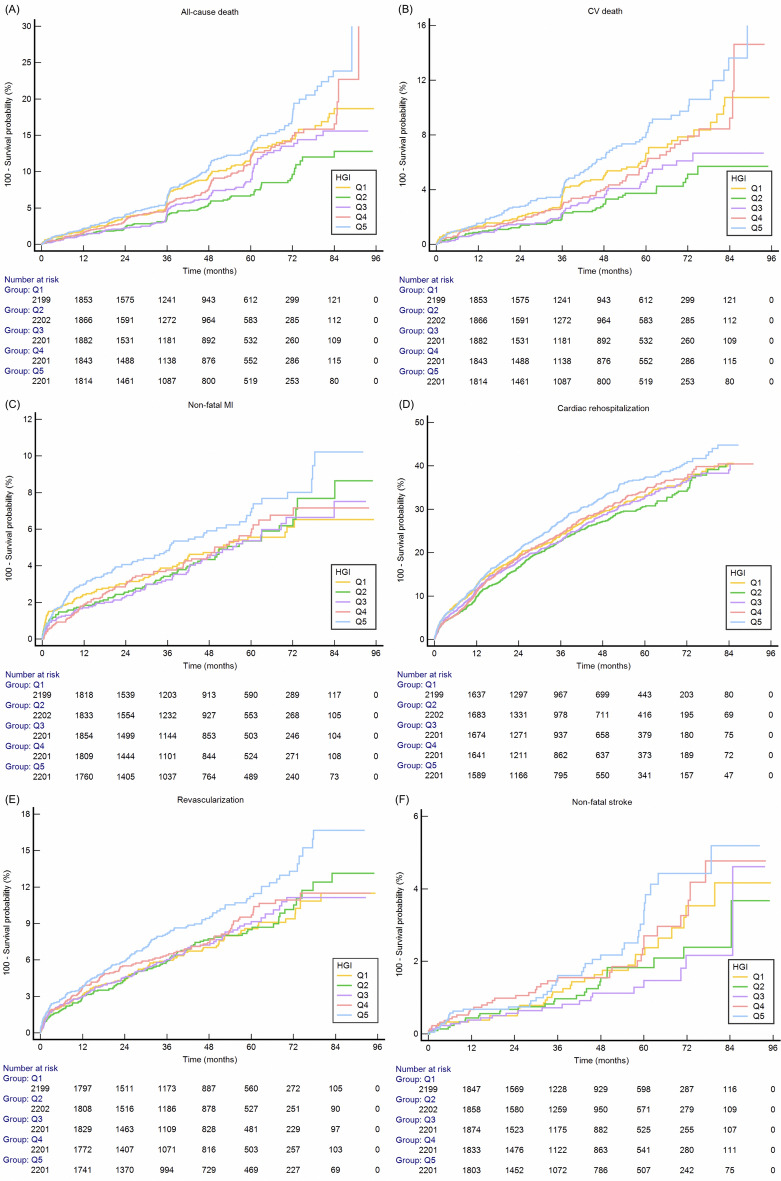
Kaplan–Meier curves for all-cause death (**A**), CV death (**B**), non-fatal MI (**C**), cardiac rehospitalization (**D**), revascularization (**E**), non-fatal stroke (**F**) of the the five quintiles. CV death, cardiovascular death; MI, myocardial infarction

**Fig. 5:**
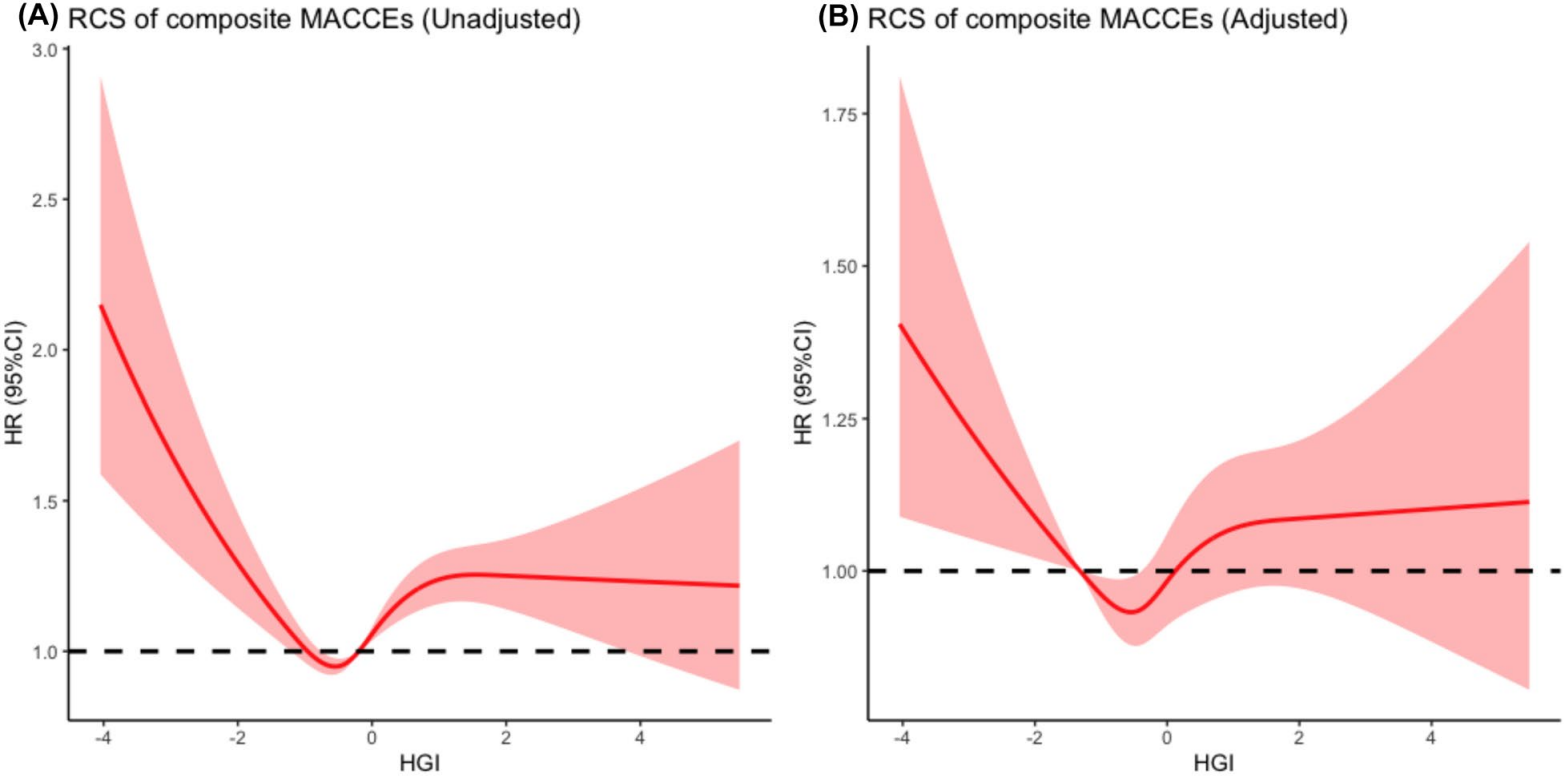
Unadjusted and adjusted RCS of HGI and the incidence of composite MACCEs. Adjusted model included age, BMI, heart rate, hypertension, previous stroke, past PCI, NSTEMI, WBC, eGFR, HDL-C, LVEF, LM/three-vessel or proximal LAD involved, and antiplatelet agents during hospitalization. *RCS* restricted cubic spline, *HGI* haemoglobin glycation index, *HR* hazard ratio, *MACCEs* major adverse cardiac and cerebral events, *BMI* body mass index, *PCI* percutaneous coronary intervention, *NSTEMI* non-ST segment elevation myocardial infarction, *WBC* white blood cells, *eGFR* estimated glomerular filtration rate, *HDL-C* high-density lipoprotein cholesterol, *LVEF* left ventricular ejection fraction, *LM* left main vessel, *LAD* left anterior descending artery

The incidence of composite MACCEs was calculated (Table 2). MACCEs occurred in 3298 (30.0%) patients [784 (7.1%) all-cause death, 420 (3.8%) CV death, 457 (4.2%) nonfatal MI, 164 (1.5%) nonfatal stroke, 2638 (24.0%) cardiac rehospitalization, 739 (6.7%) revascularizations]. Low and high HGI leaded to increased risk of all-cause death, CV death, and composite MACCEs significantly increased along with HGI levels (p < 0.001), while patients with moderate HGI (Q2: −0.491 (−0.663, −0.343)) presented the lowest rate of the above outcomes. During the median of 36.5 months of follow-up, Kaplan–Meier analysis of event-free survival indicated that there was a significant difference of survival rate among HGI groups (Figs. 3 and 4). Cox regression analyses and predictors for subvarieties of MACCEs is presented (Table 3). Univariate analysis found that the predictors associated with MACCEs occurrence were HGI, age, hypertension, diabetes, previous stroke/MI, past PCI/CABG, BMI, blood pressure, heart rate, diagnosis with NSTEMI, UA, angiotensin-converting enzyme inhibitor/angiotensin receptor blocker (ACEI/ARB) usage at admission, laboratory data including WBC, haemoglobin, Hs-CRP, RBG at admission, FPG, HbA1c, albumin, creatinine, eGFR, TC, LDL-C, HDL-C, left ventricular ejection fraction, LM/three-vessel or proximal LAD involved, medication during hospitalization including antiplatelet agents, ACEI/ARB and statins, alpha-glucosidase inhibitor and insulin usage (*P* < 0.05). After adjusting for confounding factors, multivariate Cox proportional hazards regression analysis indicated that age, hypertension, previous stroke, past PCI, BMI, heart rate, NSTEMI, WBC, eGFR, HDL-C, LVEF, LM/three-vessel or proximal LAD involved, and antiplatelet agents during hospitalization independently predicted the incidence of MACCEs in ACS patients. Finally, competing risk regression analysis was employed to compare the endpoints in different groups. The results indicate that the cumulative occurrence of CV death, nonfatal MI, revascularization, and nonfatal MACCEs were significantly correlated with HGI levels on unadjusted competing risk modelling. Notably, after adjusting for confounding factors, the multivariate-adjusted hazard ratio (HR) also increased with increasing HGI for CV death (*P* < 0.05). It is reported that patients with HGI of Q2-Q3 may suffer the lowest incidence of CV death and nonfatal stroke [CV death: Q2: 0.547 (0.403–0.742); Q3: 0.466 (0.340,0.640); nonfatal stroke: Q2: 0.512 (0.305,0.860); Q3: 0.625 (0.387,1.011)] (Table 4). To further investigate this issue, RCS were employed to analyze the relationship between HGI and the incidence of MACCEs. An HGI between −1.32 and 0.12 positively impacted the composite MACCEs after adjusting for confounding factors (χ^2^ = 12.7, *P* = 0.005) (Fig. 5). Similar results were also found for all-cause death (HGI between −1.32 and 0.46) (χ^2^ = 25.3, *P* < 0.001) and CV death (HGI between −1.32 and −0.08) (χ^2^ = 11.9, *P* = 0.008) (Additional file 1: Figure S1).

**Table 2:** Clinical outcomes

Variable	Total population	Quintile 1	Quintile 2	Quintile 3	Quintile 4	Quintile 5	p value
	n = 11004	n = 2199	n = 2202	n = 2201	n = 2201	n = 2201	
All-cause death	784 (7.1%)	183 (8.3%)	112 (5.1%)	132 (6.0%)	162 (7.4%)	195 (8.9%)	< 0.001
CV death	420 (3.8%)	99 (4.5%)	58 (2.6%)	65 (3.0%)	84 (3.8%)	114 (5.2%)	< 0.001
Non-fatal MI	457 (4.2%)	91 (4.1%)	87 (4.0%)	81 (3.7%)	87 (4.0%)	111 (5.0%)	0.194
Cardiac rehospitalization	2638 (24.0%)	546 (24.8%)	498 (22.6%)	514 (23.4%)	520 (23.6%)	560 (25.4%)	0.176
Revascularization	739 (6.7%)	137 (6.2%)	142 (6.4%)	138 (6.3%)	148 (6.7%)	174 (7.9%)	0.150
Non-fatal stroke	164 (1.5%)	34 (1.5%)	28 (1.3%)	22 (1.0%)	38 (1.7%)	42 (1.9%)	0.098
Composite MACCEs	3298 (30.0%)	687 (31.2%)	606 (27.5%)	619 (28.1%)	662 (30.1%)	724 (32.9%)	< 0.001

*CV* cardiovascular, *MI* myocardial infarction, *MACCEs* Major Adverse Cardiac and Cerebrovascular events

**Table 3:** Independent predictors of composite MACCEs

Variable	Univariate		Multivariate	
	HR (95% CI)	*p* value	Adjusted HR (95% CI)	*p* value
HGI group		< 0.001		0.012
Q5	Ref.	Ref.	Ref.	Ref.
Q4	0.864 (0.778, 0.960)	0.007	0.889 (0.799, 0.989)	0.030
Q3	0.789 (0.708, 0.878)	< 0.001	0.861 (0.771, 0.960)	0.007
Q2	0.746 (0.670, 0.831)	< 0.001	0.835 (0.747, 0.932)	0.001
Q1	0.858 (0.773, 0.952)	0.004	0.929 (0.833, 1.035)	0.173
Age, years	1.020 (1.017, 1.024)	< 0.001	1.011 (1.006, 1.016)	< 0.001
Male gender	0.985 (0.917, 1.058)	0.686		
Medical history				
Current/ex-Smoker	1.018 (0.950, 1.090)	0.614		
Hypertension	1.239 (1.146, 1.339)	< 0.001	1.109 (1.022, 1.204)	0.013
Diabetes	1.309 (1.222, 1.403)	< 0.001		
Dyslipidemia	0.972 (0.907, 1.041)	0.415		
Previous Stroke	1.375 (1.265, 1.495)	< 0.001	1.208 (1.109, 1.317)	< 0.001
Previous MI	1.369 (1.239, 1.512)	< 0.001		
Past PCI	1.372 (1.256, 1.499)	< 0.001	1.268 (1.157, 1.389)	< 0.001
Past CABG	1.232 (1.005, 1.511)	0.045		
Physical examination				
BMI, kg/m^2^	0.984 (0.975, 0.994)	0.001	0.987 (0.977, 0.997)	0.012
SBP, mmHg	1.003 (1.002, 1.005)	< 0.001		
DBP, mmHg	0.996 (0.993, 0.999)	0.01		
Heart rate, bpm	1.008 (1.005, 1.010)	< 0.001	1.004 (1.002, 1.007)	0.001
Clinical presentation				
STEMI	1.042 (0.954, 1.139)	0.357		
NSTEMI	1.407 (1.295, 1.530)	< 0.001	1.205 (1.068, 1.358)	< 0.001
UAP	0.779 (0.726, 0.835)	< 0.001		
Medication on admission				
Antiplatelet agent	1.070 (0.998, 1.148)	0.057		
ACEI/ARB	1.107 (1.031, 1.189)	0.005		
Beta-blocker	0.989 (0.911, 1.073)	0.783		
Statins	0.983 (0.912, 1.059)	0.649		
Laboratory data				
WBC, 10^9^/L	1.024 (1.010, 1.037)	0.001	1.020 (1.004, 1.036)	0.008
Hemoglobin, g/L	0.992 (0.990, 0.994)	< 0.001		
Hs-CRP, mg/L	1.013 (1.010, 1.016)	< 0.001		
RBG at admission, mmol/L	1.023 (1.014, 1.032)	< 0.001		
FPG, mmol/L	1.048 (1.034, 1.062)	< 0.001		
HbA1c,%	1.077 (1.053, 1.102)	< 0.001		
Albumin, g/L	0.954 (0.946, 0.962)	< 0.001		
Creatinine, umol/L	1.002 (1.001, 1.002)	< 0.001		
eGFR, ml/min/1.73m^2^	0.988 (0.987, 0.990)	< 0.001	0.993 (0.991, 0.995)	< 0.001
TC, mmol/L	0.949 (0.918, 0.982)	0.002		
TGs, mmol/L	0.986 (0.958, 1.016)	0.361		
LDL-C, mmol/L	0.952 (0.909, 0.997)	0.035		
HDL-C, mmol/L	0.745 (0.653, 0.850)	< 0.001	0.809 (0.681, 0.962)	0.002
Echocardiography				
LVEF	0.107 (0.076, 0.148)	< 0.001	0.240 (0.167, 0.346)	< 0.001
Angiography findings				
LM/three-vessel	1.233 (1.150, 1.321)	< 0.001	1.208 (1.117, 1.307)	< 0.001
Proximal LAD	0.917 (0.855, 0.983)	0.014	0.914 (0.848, 0.986)	0.019
Medication during hospitalization				
PCI/CABG	1.058 (0.987, 1.133)	0.111		
Antiplatelet agent	0.774 (0.679, 0.882)	< 0.001	0.806 (0.693, 0.937)	0.020
ACEI/ARB	1.096 (1.022, 1.174)	0.010		
Beta-blocker	1.040 (0.965, 1.121)	0.301		
Statins	0.842 (0.756, 0.937)	0.002		
Hypoglycemic agents				
Alpha-glucosidase inhibitor	1.198 (1.108, 1.296)	< 0.001		
Metformin	1.056 (0.954, 1.168)	0.293		
Sulfonylurea	0.994 (0.876, 1.128)	0.925		
DPP-4i	1.165 (0.437, 3.107)	0.760		
Insulin	1.352 (1.208, 1.512)	< 0.001		
Insulin sensitizer	1.014 (0.814, 1.263)	0.901		

*MACCEs* major adverse cardiac and cerebral events, *HGI* hemoglobin glycation index, *MI* myocardial infarction, *PCI* percutaneous coronary intervention, *CABG* Coronary Artery Bypass Grafting, *BMI* body mass index, SBP systolic blood pressure, *DBP* diastolic blood pressure, *STEMI*ST-elevated myocardial infarction, *NSTEMI* non-ST elevated myocardial infarction, *UAP* unstable angina pectoris, *ACEI* angiotensin-converting enzyme inhibitors, *ARB* angiotensin receptor blockers, *WBC* white blood cells, *Hs-CRP* Hypersensitive c-reactive protein, *RBG* random blood glucose, *FPG* fast plasma glucose, *eGFR* estimated glomerular filtration rate, *TC* total cholesterol, *TGs* triacylglycerol, *LDL-C* low density lipoprotein cholesterol, *HDL-C* high density lipoprotein cholesterol, *LVEF* left ventricular ejection fraction, *LM* left main vessel, *LAD* left anterior descending artery, *DPP-4i* dipeptidyl peptidase-4 inhibitors

**Table 4:** Competing risk model of clinical outcomes

	Unadjusted HR (95% CI)	p value	Adjusted HR (95% CI)	p value
CV death				
Q5	Ref.		Ref.	
Q4	0.703 (0.530, 0.931)	0.014	0.655( 0.486, 0.881)	0.005
Q3	0.547 (0.403, 0.742)	< 0.001	0.606 (0.438, 0.838)	0.002
Q2	0.466 (0.340, 0.640)	< 0.001	0.585 (0.422, 0.811)	0.001
Q1	0.792 (0.605, 1.037)	0.089	0.811 (0.605, 1.087)	0.160
Non-fatal MI				
Q5	Ref.		Ref.	
Q4	0.736 (0.551, 0.983)	0.038	0.788 (0.587, 1.057)	0.112
Q3	0.689 (0.513, 0.924)	0.013	0.806 (0.599, 1.087)	0.157
Q2	0.732 (0.549, 0.975)	0.033	0.851 (0.636, 1.139)	0.278
Q1	0.746 (0.560, 0.993)	0.045	0.772 (0.576, 1.036)	0.084
Cardiac rehospitalization				
Q5	Ref.		Ref.	
Q4	0.895 (0.792, 1.010)	0.072	0.942 (0.833, 1.064)	0.336
Q3	0.868 (0.768, 0.980)	0.023	0.949 (0.839, 1.074)	0.411
Q2	0.823 (0.729, 0.931)	0.002	0.894 (0.791, 1.012)	0.077
Q1	0.892 (0.791, 1.007)	0.065	0.955 (0.843, 1.081)	0.462
Revascularization				
Q5	Ref.		Ref.	
Q4	0.808 (0.647, 1.008)	0.059	0.907 (0.725, 1.135)	0.393
Q3	0.747 (0.596, 0.936)	0.011	0.890 (0.709, 1.117)	0.314
Q2	0.761 (0.609, 0.950)	0.016	0.854 (0.682, 1.069)	0.168
Q1	0.712 (0.568, 0.893)	0.003	0.746 (0.591, 0.941)	0.013
Non-fatal stroke				
Q5	Ref.		Ref.	
Q4	0.833 (0.532, 1.305)	0.425	0.924 (0.587, 1.455)	0.734
Q3	0.512 (0.305, 0.860)	0.011	0.594 (0.350, 1.007)	0.053
Q2	0.625 (0.387, 1.011)	0.055	0.713 (0.438, 1.160)	0.173
Q1	0.709 (0.447, 1.126)	0.145	0.762 (0.479, 1.214)	0.253
Non-fatal MACCEs				
Q5	Ref.		Ref.	
Q4	0.885 (0.790, 0.992)	0.037	0.930 (0.829, 1.044)	0.219
Q3	0.821 (0.731, 0.923)	0.001	0.904 (0.803, 1.017)	0.093
Q2	0.794 (0.707, 0.892)	< 0.001	0.875( 0.779, 0.984)	0.026
Q1	0.890 (0.794,0.997)	0.044	0.946 (0.842, 1.062)	0.346

*CV* cardiovascular, *MI* myocardial infarction, *MACCEs* Major Adverse Cardiac and Cerebrovascular events

### Independent association of HGI with MACCEs in different subgroups

**Fig. 6:**
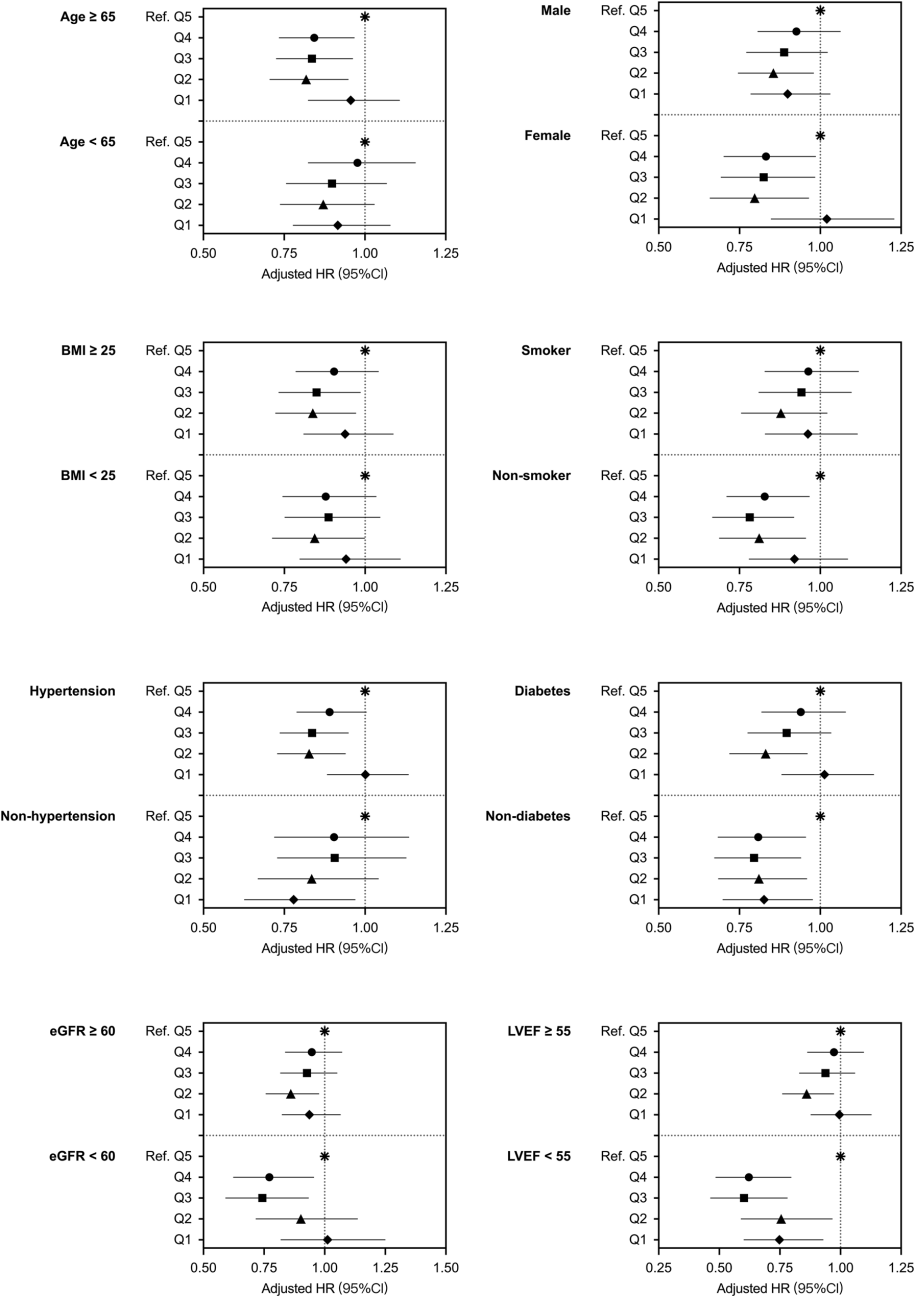
Forest plot of composite MACCEs according to different subgroups. Adjusted model included age, BMI, heart rate, hypertension, previous stroke, past PCI, NSTEMI, WBC, eGFR, HDL-C, LVEF, LM/three-vessel or proximal LAD involved, and antiplatelet agents during hospitalization. *HR* hazard ratio, *MACCEs* major adverse cardiac and cerebral events, *BMI* body mass index, *PCI* percutaneous coronary intervention, NSTEMI non-ST segment elevation myocardial infarction, *WBC* white blood cells, *eGFR* estimated glomerular filtration rate, *HDL-C* high-density lipoprotein cholesterol, *LVEF* left ventricular ejection fraction, *LM* left main vessel, *LAD* left anterior descending artery, *Ref*. reference(Q5, 0.485 ≤ HGI < 7.875)

Subgroup analysis was carried out according to age, sex, BMI, smoker, hypertension, diabetes, eGFR, and LVEF, demonstrating a predictive effect of HGI on MACCEs in many subgroups (Fig. 6). For patients aged ≥ 65 years, moderate HGI (Q2, Q3, Q4) usually comes with a lower incidence of MAACEs. Male patients with Q2 HGI and female patients with Q2-Q4 HGI suffered a lower risk of MACCEs. For patients with BMI ≥ 25 and hypertension, HGI within Q2-Q3 was correlated with a lower incidence of MACCEs. Patients with LVEF < 55 or without diabetes had a lower risk of MACCEs in the Q1-Q4 HGI groups than in the Q5 HGI group.

## Discussion

This study aimed to elucidate the predictive effects of HGI levels on the outcomes in ACS patients with or without diabetes, to the best of our knowledge. The main findings include the following: (1) A U-shaped relationship was reported between HGI levels and incidence of MACCEs. Both lower and higher HGI could cause an increased risk of poor outcomes in ACS patients. This finding was consistent with Yuesong’s work in diabetic patients with ischaemic stroke [15]. They identified that in diabetic patients coexisting with ischaemic stroke, low HGI and high HGI contributed to an increased risk of stroke recurrence and poor outcome. (2) The predictive effect of HGI on MACCEs is powerful in Q2-Q3 (−0.491 to −0.196). (3) Several clinical factors, such as age, hypertension, previous stroke, past PCI, BMI, and heart rate, independently predicted the incidence of MACCEs in ACS patients.

There are no clear mechanisms of the variation between the actual and predicted levels oof HbA1c. HbA1c is glycated haemoglobin formed by an intracellular nonenzymatic reaction, while FPG reflects the plasma glucose condition [16]. Considering this situation, lower erythrocyte turnover rates may contribute to the accumulation of HbA1c [17]. According to existing evidence, glycation is a complex biological process affected by various factors, factors that influence intracellular glucose concentrations or nonenzymatic haemoglobin glycation (such as the intracellular pH value) may also affect the degree of haemoglobin glycation [16, 17]. From this perspective, all factors regulated glucose metabolism may contribute to the individual HGI variations.

A large body of evidence has focused on the association of HGI with clinical situations [13, 18, 19]. In the Diabetes Control and Complications Trial (DCCT), Twomey et al. found that in patients with type 1 diabetes, the increased rate of retinopathy and nephropathy usually comes after higher HGI [13]. In addition, the Control Cardiovascular Risk in Diabetes (ACCORD) trial [18] reported that patients with low and moderate HGI levels could benefit from intensive treatment, which is not observed in high HGI group. Unlike the linear relation between HGI and vascular complications reported in previous studies, our study reported a U-shaped association of HGI with the prognosis of ACS patients. Both patients with low and high HGI had poorer prognoses than those with moderate HGI. One potential explanation is stress hyperglycemia [20], a feature of ACS. Stress hyperglycaemia could contribute to high FPG followed by low HGI, but more research is needed to confirm this point.

To better understand the predictive power of HGI for ACS, this study analyzed the correlation between HGI and each type of MACCE and found that HGI was closely related to CV death. In addition, we determined the predictive value of HGI on the composite of MACCEs in different subgroups, such as sex, age, and medical history, indicating that HGI is a good predictor for MACCEs. There is limited evidence showing clinical factors affecting the effect of the HGI value. Di-Shuang et al. [21]. found that higher HGI increased the incidence of hepatic steatosis when adjusted for age, sex, and BMI. Several studies also reported a close relationship between HGI and sex [18, 22], while others did not show any sex differences in HGI [23, 24]. In the future, more research is required to assess the findings. Although HGI is a complex parameter to measure the differences between predict and actual levels of glycation of haemoglobin, current evidence indicated that HGI is helpful to evaluate the prognosis of ACS patients, which may help to develop personalized treatment strategies.

In addition, Table 1 demonstrated that there is significant difference of the usage of ACEI/ARB, statin among different HGI groups. This promoted us to investigate the interactions between drugs and HGI levels. Currently, there are few studies focused on this issue. However, we could study from some indirect evidence. A study focused on the efficacy of HGI on non-diabetes patients, and it came out that high HGI were likely to come with obese, higher levels of TG and lower levels of HDL-C [19]. From this aspect, statin treatment may contribute to lower HGI levels. Various researches have reported the efficacy of ACEI/ARBs or SGLT2 inhibitors in diabetes. HGI and HbAc1 shared some commons, indicating that these agents may also influence HGI levels. To further digging the relationship between HGI levels and drug usage.

### Limitations

First, although this study included a large sample size, there were still bias due to the single-center and retrospective design issue. Second, laboratory parameters were only measured once during the hospitalization period, which could cause potential bias. Third, we enrolled patients with or without diabetes, which may weaken the confidence of our findings. In the following step, we will enroll ACS patients with diabetes to further investigate the predictive value of HGI. Finally, more prospective cohort studies are necessary to confirm our results.

## Conclusion

Conclusively, this study firstly demonstrated the relationship between hemoglobin glycation index and outcomes in patients diagnosed with ACS. Both low HGI and high HGI was reported to attribute higher risk of poor prognosis in ACS patients compared with moderate HGI.

## Supplementary Information

**Additional file 1: Figure S1.** Adjusted RCS of HGI and the incidence of all-cause death (A), CV death (B), non-fatal MI (C), cardiac rehospitalization (D), revascularization (E), non-fatal stroke (F). Adjusted model included age, BMI, heart rate, hypertension, previous stroke, past PCI, NSTEMI, WBC, eGFR, HDL-C, LVEF, LM/three-vessel or proximal LAD involved, and antiplatelet agents during hospitalization. RCS, restricted cubic spline; HGI, haemoglobin glycation index; HR, hazard ratio; MACCEs, major adverse cardiac and cerebral events; CV death, cardiovascular death; MI, myocardial infarction; BMI, body mass index; PCI, percutaneous coronary intervention; NSTEMI, non-ST segment elevation myocardial infarction; WBC, white blood cells; eGFR, estimated glomerular filtration rate; HDL-C, high-density lipoprotein cholesterol; LVEF, left ventricular ejection fraction; LM, left main vessel; LAD, left anterior descending artery.

## Data Availability

The datasets generated and/or analyzed during the current study are not publicly available due to the provisions of the CBD Bank but are available from the corresponding author on reasonable request.

## Abbreviations

HGI: Haemoglobin glycation index
ACS: Acute coronary syndrome
MACCEs: Major adverse cardiac and cerebral events
HR: Hazard ratio
CV death: Cardiovascular death
PCI: Percutaneous coronary intervention
BMI: Body mass index
NSTEMI: Non-ST segment elevation myocardial infarction
WBC: White blood cells
eGFR: Estimated glomerular filtration rate
HDL-C: High-density lipoprotein cholesterol
LVEF: Left ventricular ejection fraction
LM: Left main vessel
LAD: Left anterior descending artery
DM: Diabetes mellitus
HbA1c: Haemoglobin A1c
FPG: Fast plasma glucose
CBD: Center of Beijing Friendship Hospital Database
RBG: Random blood glucose
OGTT: Oral glucose tolerance test
TC: Total cholesterol
LDL-C: Low-density lipoprotein cholesterol
TG: Triglyceride
STEMI: ST segment elevation myocardial infarction
UA: Unstable angina
SD: Standard deviation
IQR: Interquartile range
CIs: Confidence intervals
RCS: Restricted cubic spline
CABG: Coronary Artery Bypass Grafting
ACEI: Angiotensin-converting enzyme inhibitors
ARB: Angiotensin receptor blockers
Hs-CRP: Hypersensitive c-reactive protein
DCCT: Diabetes Control and Complications Trial
ACCORD: Control Cardiovascular Risk in Diabetes trial
