# Supplementary figures and images for: Association of haemoglobin glycation index with outcomes in patients with acute coronary syndrome: results from an observational cohort study in China
Source: Diabetol Metab Syndr. 2022 Oct 31;14:162. doi: 10.1186/s13098-022-00926-6 (PMC9620631; doi:10.1186/s13098-022-00926-6)

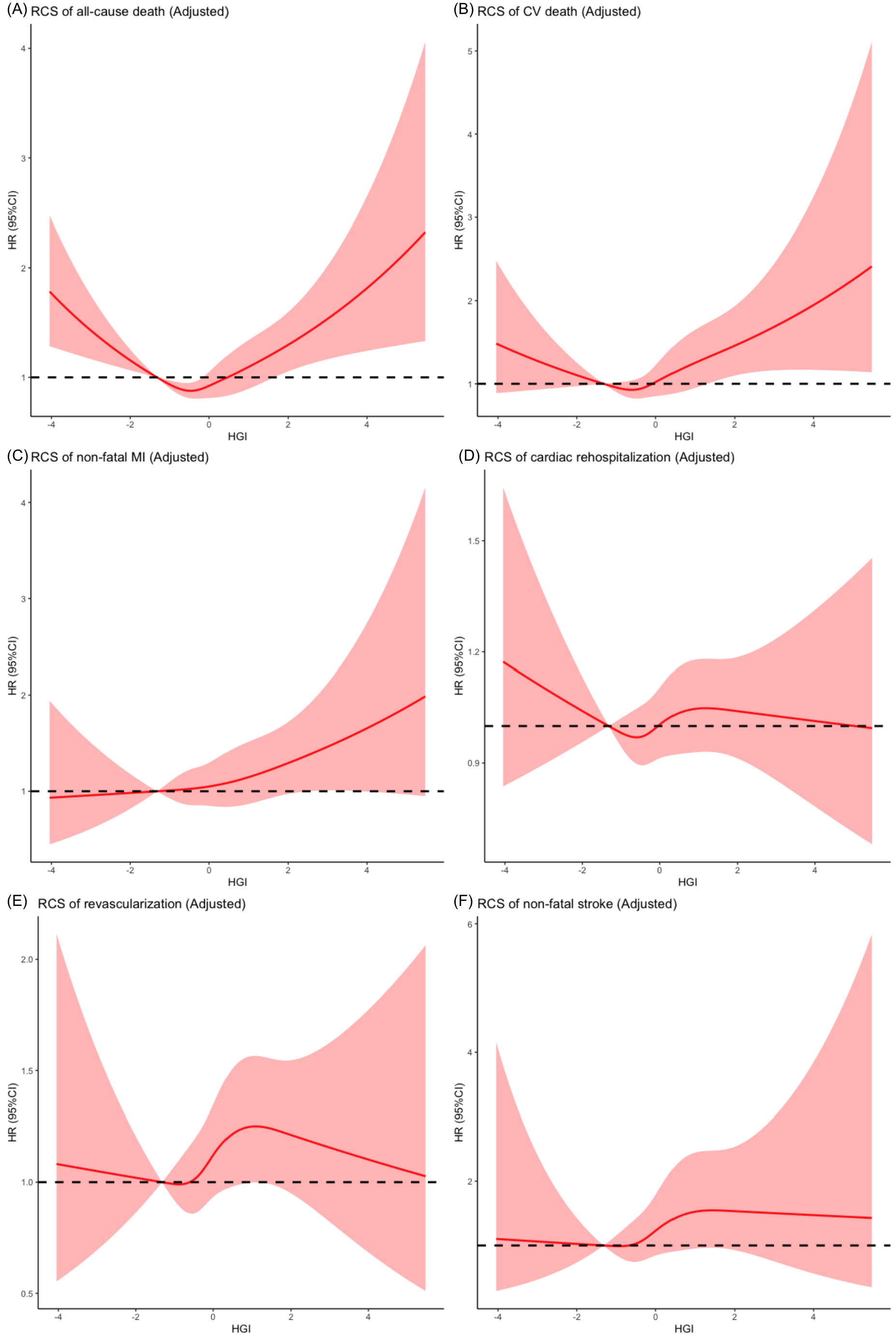

Supplement: Supplementary file 1 — Additional file 1: Figure S1. Adjusted RCS of HGI and the incidence of all-cause death (A), CV death (B), non-fatal MI (C), cardiac rehospitalization (D), revascularization (E), non-fatal stroke (F). Adjusted model included age, BMI, heart rate, hypertension, previous stroke, past PCI, NSTEMI, WBC, eGFR, HDL-C, LVEF, LM/three-vessel or proximal LAD involved, and antiplatelet agents during hospitalization. RCS, restricted cubic spline; HGI, haemoglobin glycation index; HR, hazard ratio; MACCEs, major adverse cardiac and cerebral events; CV death, cardiovascular death; MI, myocardial infarction; BMI, body mass index; PCI, percutaneous coronary intervention; NSTEMI, non-ST segment elevation myocardial infarction; WBC, white blood cells; eGFR, estimated glomerular filtration rate; HDL-C, high-density lipoprotein cholesterol; LVEF, left ventricular ejection fraction; LM, left main vessel; LAD, left anterior descending artery. [file 13098_2022_926_MOESM1_ESM.jpg]
